# Avoidant romantic attachment in adolescence: Gender, excessive internet use and romantic relationship engagement effects

**DOI:** 10.1371/journal.pone.0201176

**Published:** 2018-07-27

**Authors:** Vasileios Stavropoulos, Stefanos Mastrotheodoros, Tyrone L. Burleigh, Nicole Papadopoulos, Rapson Gomez

**Affiliations:** 1 School of Psychology, University of Athens, Athens, Attica, Greece; 2 Cairnmillar Institute, School of Psychology, Melbourne, Victoria, Australia; 3 Research Centre Adolescent Development, Child and Adolescent Studies, Utrecht University, Utrecht, the Netherlands; 4 School of Psychology, Federation University, Mount Helen, Victoria, Australia; 5 School of Psychology, Deakin University, Burwood, Victoria, Australia; Pace University, UNITED STATES

## Abstract

Romantic development is a distinctive characteristic of puberty. However, a significant proportion of adolescents present with avoidant romantic attachment (ARA) tendencies, which have significant impact on their general adaptation. ARA variations have been suggested in relation to age, gender, engagement with a romantic partner and Excessive Internet Use (EIU) behaviours. In this longitudinal, two-wave study of a normative sample of 515 Greek adolescents at 16 and 18 years, ARA was assessed with the relevant subscale of the Experiences in Close Relationships-Revised and EIU with the Internet Addiction Test. A three-level hierarchical linear model found ARA tendencies to decrease between 16 and 18 while engagement in a romantic relationship and EIU were associated with lower and higher ARA tendencies respectively. Gender did not differentiate ARA severity either at the age of 16 or its changes over time. Results highlight the need of adopting a longitudinal-contextualized approach and provide implications for prevention and intervention initiatives in relation to the romantic development of adolescents.

## Introduction

Engagement in romantic relationships during adolescence is important for individual development and well-being. It has been positively associated with the formation of personal identity, adaptability to change, self-esteem, social competence, and even scholastic achievement and career planning [1,2]. However, several contextual (e.g. parenting style, parent related role modelling effects, peer influences due to social compliance) and individual factors, such as insecure attachment styles, may hinder engagement in romantic relationships during adolescence [3]. Insecure attachment styles have been repeatedly acknowledged as pivotal intra-individual predictors of the quality of engagement in romantic relationships over the lifespan in general and during adolescence in particular [4]. These important developmental aspects are inevitably affected by the expansion of Internet access among modern adolescents. In particular, adolescents appear to prefer cyber-relationship applications [5], such as online chatrooms and social networking sites, which may often accommodate online romantic interactions.

Literature has consistently highlighted two major dimensions of insecure attachment, the anxious and the avoidant [6]. The anxious dimension has been defined as a combination of the desire to be close with others and the worry of being abandoned, and the avoidant dimension has been featured as a fear of closeness intertwined with the tendency to avoid interpersonal dependency [6]. Although studies have concluded that both anxious and avoidant attachment tendencies are detrimental to the cognitive, behavioural, and emotional aspects of romantic engagement [4], their impact has been shown to differ [7,8]. Higher levels of anxious attachment manifestations have been associated with increased sensitivity to signs of rejection, while higher levels of avoidance have been found to prompt higher withdrawal behaviour [7]. This is concordant with recent findings noting that more avoidant individuals tend to use a repertoire of deactivating strategies (e.g., pre-emptive and post-emptive behaviours) to regulate their emotional processing [8]. In that line, comprehensive meta-analytic findings support that avoidant romantic attachment (ARA) tendencies are more negatively associated with feelings of general satisfaction, connectedness and general support in relationships in adolescence and adulthood than anxious romantic attachment tendencies [4]. The above may be particularly significant in the context of Internet use expansion among contemporary adolescents, that may encourage the substitution of face to face with online interactions [5]. For example, excessive use of the Internet may further reinforce the lower facial expressions decoding accuracy presented by more avoidant individuals [9]. Therefore, investigating factors that contribute to ARA may increase mental health and education professionals’ understanding of romantic engagement during adolescence and thus, enable them to better support it.

### Age related changes in romantic attachment during adolescence

The present study aims to examine age related factors that may potentially contribute to ARA tendencies during adolescence. Adolescence is a pivotal developmental stage [10], associated with an increase in the engagement in romantic relationships [1]. Specifically, it has been postulated that early adolescence (11–13 years) is a period when individuals experience romantic attractions and affiliations; middle adolescence (14–16 years) is a period of deeper exploration of romantic relationships, and late adolescence (16–18 years) is a period of consolidation of romantic bonds [3]. Thus, patterns of insecure romantic attachment develop during late adolescence, which has been suggested to increase the risk of poorer quality romantic relationships in adult life [3]. Given that ARA manifestations have been repeatedly linked with non-emotionally sexual and non-committed encounters, as well as risky sexual behaviour, the investigation of ARA in this stage is particularly significant [11–13].

Studies contend that ARA severity varies over time. Specifically, ARA tendencies have been found to increase after 20 years of age, particularly in males, and appear to stabilize after the age of 40 years [14]. Despite this, there has been a dearth of studies that have investigated age related changes in ARA during late adolescence. Maturational and socialization processes [15,16] occurring over the course of adolescence, may better equip individuals to address their discomfort with romantic closeness. For example, better socio-cognitive skills such as attention focusing, abstract thinking, social reasoning, and advanced thought processing may lead to a reduction in the manifestation of ARA tendencies. Furthermore, avoidant attachment has been linked with the sympathetic and the parasympathetic nervous system, which undergo significant developmental changes during adolescence and underlie reactions to discomfort and withdrawal from situations, such as those that romantic avoidance might involve [17–19]. Based on the above literature, it is thus likely, that ARA severity may gradually decrease (due to the psychological and biological maturation processes involved) during the period of late adolescence. In that line, although recent findings indicate a relative stability of attachment styles over adolescence, they contend that individuals who are not exposed to significant risk factors increase their levels of attachment security between 16 and 18 years [20,21]. These contradict findings suggesting that ARA severity progressively increases in males potentially reflecting different developmental trajectories/phases occurring during adolescence compared to emerging and middle adulthood, which need to be more carefully considered [14].

Accounting for different cultural effects on the development (progress over time) of romantic attachment is also of particular interest especially in under studied cultural populations [1]. In the present study, age-related effects on ARA severity are studied in a normative sample of Greek adolescents. Given the dearth of ARA related studies in Greece, Greek adolescents comprise a significant, from a developmental perspective [1], and concurrently understudied, from a cultural perspective, population [9,22,23]. At the same time, empirical evidence has suggested that Greek youth approach close relationships in a manner that differs, for example, from this of American youth [9,24,25]. These could indicate that Greek cultural values, involving concepts of culturally appropriate and/or accepted behaviours in romantic relationships, could differentiate ARA severity during the period of late adolescence [26]. Specifically, the higher emphasis on traditional values/constructs, such as family and marriage, that underpin romantic relationships in the Greek culture compared to other Western cultures, could uniquely affect the development of ARA behaviours [27].

### Gender influences on romantic attachment in adolescence

Whilst age related changes may be linked with intra-individual variations in ARA, gender has also been associated with ARA behaviours. Del Giudice [28] proposes that ARA may be conceived as a male related pattern of attachment that aims to reduce exposure/commitment to long-term relationships and may explain the tendency for males to be oriented to short-term relationships and sexual interactions [14]. This argument has also been reinforced by cross-sectional empirical findings [14,25]. More recently, gender differences regarding ARA have been associated with specific behavioural facets which appear to be higher in males. These include “self-reliance” (i.e., decreased emotional need for support from one's partner; lack of reliance on one’s partner as a safety net during stressful conditions) and “discomfort with closeness” (i.e., ambivalence towards emotional closeness due to a difficulty in selecting the right emotional distance in relationships) [28]. Furthermore, it has been illustrated that gender differences in ARA may vary over the lifespan [14]. Specifically, gender differences in ARA have been said to increase between 20 and 40 years of age, especially for males, and could diminish in contexts where the reproductive interests of males and females converge [14]. Additionally, gender differences in romantic attachment have been shown to decrease in student samples which may be attributed to social compliance effects [14].

The developmental period during which gender related differences in regard to ARA emerge is unclear, as is whether they are consistent across different cultural populations. Implementing longitudinal research to clarify this point is needed. This is additionally reinforced by findings that suggest gender related variations in facets of ARA across different cultural samples [28]. In particular, cross-sectional findings from cross-cultural studies indicate that Greek samples, in contrast to national samples of several other countries including Canada, USA, Chile, Argentina, the Netherlands, and the UK, do not present significant gender differences in relation to romantic attachment [25]. This could be a result of aspects involving the strong collectivistic and family orientation of the Greek culture, which may overwrite gender differences in ARA tendencies [27–29].

### Romantic involvement influences in romantic attachment in adolescence

On top of age-, and gender-related influences on romantic attachment, there is strong evidence supporting the importance of involvement in romantic relationships during adolescence [1,30]. Some studies indicate that involvement in a romantic relationship during adolescence may act as a protective factor for adolescents’ general adaptation precipitating higher levels of psychological wellbeing [31,32]. In contrast, other studies demonstrate that the engagement in romantic relationships in adolescence may be associated with higher delinquent behaviours, signs of depression, and suicidal behaviour [33,34]. Overall, researchers conclude that engagement in a romantic relationship in adolescence can function both as a risk and as an opportunity depending on the qualities of the romantic interaction [35]. Collins et al. (2009) suggest that romantic relationships that are lengthier, more committed, and of a particular intensity (i.e., solid frequency and have a structure/specific pattern of meetings and interactions) tend to function positively for adolescent development and adult romantic involvement [1].

Considering the influences of romantic involvement on ARA in particular, studies have supported that negative past experiences of romantic relationships may shape internal representations and perceptions of relationships which could later induce ARA [36]. In line with these findings, it was revealed that the impact of a negative previous relationship is the most common reason for exhibiting ARA behaviours [37]. However, the association between ARA tendencies and involvement in romantic relationships appears to be bidirectional with research confirming the effect of ARA tendencies on both the engagement and the quality of romantic relationships over the life span [4,38].

Despite these findings, there is a paucity of longitudinal studies examining the potential protective effects of lengthier, more committed and of a particular intensity experiences of romantic relationships on ARA tendencies. This is important given that romantic relationship quality in late adolescence predicts romantic relationship quality in emerging adulthood [39]. Based on the broader concept of Fraley’s “prototype model,” which supports that earlier attachment experiences continue to exert influence over the years, a positive romantic involvement at the age of 16 may reduce ARA tendencies at the age of 18 [40]. This expectation appears to be in consensus with recent findings which support the prototype hypothesis and the relative stability of attachment styles over adolescence and adulthood [20]. The present study aims to contribute to the existing knowledge by examining the potential effect of the experience of more stable and lengthy romantic relationships on ARA tendencies during late adolescence.

### Internet use and romantic attachment

In conjunction with the age related and individual (e.g., gender, involvement in a romantic relationship) factors highlighted above, Internet use has been suggested to significantly contribute to romantic behaviours of contemporary adolescents [41]. The Internet has been shown to be used by adolescents to address several developmental tasks including identity development, nurturing of friendship networks as well as their romantic relationships [41]. Despite the general agreement considering the increased influence of the Internet in the romantic life of adolescents, there have been inconsistent findings regarding whether its contribution is negative or positive [42–44]. There have been studies suggesting that more time spent online predicts increased sexual/romantic experience in adolescence and promotes the formation of romantic relationships, especially among adolescents with same-sex attraction [43,44]. Other research findings have suggested that Internet use is associated with impulsive and risky sexual behaviour among both adolescents and young adults (i.e., multiple encounters) [45,46].

Two different theoretical hypotheses may explain these contradictory findings. The *“rich-get-richer”* hypothesis assumes that adolescent internet users, who already are competent in romantic relationships and interactions, may use the medium for additional opportunities in a way that further promotes their romantic life [43]. In contrast, the *“social compensation hypothesis”* suggests that adolescents with high levels of romantic avoidance may use the internet to counterbalance their difficulties, thus being less motivated to develop the skills required in offline romantic relationships [47]. On this basis, comprehensive literature reviews suggest that the effect of internet use on romantic interactions, independent of the age of the user, can be both *“debilitating and/or deliberating”* depending on specific qualities such as time spent online, type of motivation, individual differences regarding the users’ profiles, and the applications used [48].

In relation to ARA in particular, several studies have reported associations between avoidant attachment and Excessive Internet Use (EIU) [49]. EIU is described as a problematic preoccupation with the Internet that may range in severity and causes psychological and social impairments and/or distress [50]. Cyber-relationships, over both adolescence and adulthood, eliminate the risks of face-to-face (FtF) closeness, thus providing a safer place for romantic experimentation, which may encourage avoidance [48]. EIU may therefore reinforce avoidance and loneliness in offline relationships across both adolescents and adults, since users progressively become more dependent on the flexibility and convenience of cyber-interactions, that may involve anonymous, accessible and affordable interactions, without developing the FtF social skills required for romantic intimacy in real life [48,51]. This hypothesis is in agreement with research indicating that an individual’s behaviours are calibrated by functionality requirements according to the conditions or the context one is exposed to. These behavioural patterns initiate as conditional adaptations that become more permanent the longer the person, irrespective of age, is exposed to the effects of a specific context -in this case the internet context [52,53]. Accordingly, one could assume that EIU may negatively influence ARA tendencies among adolescents, fostering fear of closeness and withdrawal from offline romantic interactions [51]. This is further reinforced by evidence indicating that higher Internet use in adolescence is associated with the initiation of sexual activity prior to the involvement in a romantic relationship [52], which interestingly has been linked to ARA tendencies in young adulthood [14]. However, the current body of literature has approached the association between EIU and ARA in the opposite way, and has specifically illustrated the causal contribution of avoidant tendencies in the development of EIU across a range of adolescent and adult samples [54,55]. Not surprisingly, adolescent EIU has been suggested to potentially function as a form of avoidant coping to address interpersonally stressful situations [56]. Furthermore, avoidant adolescents have been assumed to use the internet as a way to cope with developmental challenges, such as engaging in real-life romantic relationships [48,57]. In this context, the present study aims to contribute to extant knowledge on the topic by longitudinally examining the potential risk effect of EIU on ARA during the period of late adolescence.

### The current study

This study adopted a dimensional (continuum of minimum to maximum) conceptualization of ARA [58] that is different from the older typological approach which suggested the assignment of individuals to distinct attachment categories [59]. This enabled the examination of the whole spectrum of variations of ARA manifestations [60] and is supported by empirical findings indicating a wide range of severity of ARA tendencies [14]. This dimensional approach on attachment has been widely followed by international researchers and its measurements have been validated in Greek populations, such as the sample used in the present study [14,27,61].

To study the whole range of variations of ARA tendencies, theoretical elements of the risk and resilience developmental framework [62] were integrated with concepts and findings of the field of romantic attachment studies [1,14,28,63–65]. The risk and resilience theoretical framework perceives behaviours (i.e. ARA) as constantly varying on a continuum because of the interplay of age, individual and contextual risks and resources [62]. To appropriately investigate the effects and the interactions of risks and resources across each of the levels involved (age related changes, individual and contextual), this framework is best employed using multiple levels of analyses that disentangle the effects related to the developmental stage, the person and the person’s context [62]. Thus, the risk and resilience framework theoretically integrates the independent variables of the current work. Subsequently, the present research model is person focused in a way that captivates diverse and subtle trajectories in ARA development during late adolescence. This kind of information is valuable for both scientists and practitioners, as it moves from describing relationships between abstract psychological constructs to more specific and “person-centred” research findings [66,67]. Such findings could inform the guidelines for more effective prevention and intervention initiatives considering ARA behaviours during adolescence.

Accordingly, the present longitudinal study examined a sample of Greek adolescents (assessed at 16 and again at 18 years of age) to determine the effects of individual level risks and resources in regard to intra-individual and inter-individual variations of ARA tendencies. Three levels of analysis were applied: Level 1 was used for the temporal factors (i.e., age related changes in ARA), level 2 was used to study two individual level risks (i.e., being a male and presenting higher EIU behaviours) and an individual resource (i.e., involvement in a romantic relationship), whilst level three controlled for random effects due to the nesting of the participants in specific classrooms. Based on relevant literature, the following specific research hypotheses were examined:

*H*_*1*_: ARA will decrease between 16 and 18 years and this may not differentiate across genders. Maturational, physiological and socialization processes unfolding during adolescence [15,16,68], would enable individuals to become gradually better at coping with their romantic closeness stressors reducing ARA tendencies severity. Furthermore, effects related to the strong collectivistic and family orientation (which is intertwined with a highly-committed perception of romantic relationships) of the Greek culture may potentially overwrite gender differences in ARA tendencies [27,28,29]. This is in consensus with previous studies which have suggested that gender specific ARA variations tend to decrease in contexts where the reproductive interests of males and females converge [14,28].

*H*_*2*_: It is hypothesized that involvement in a romantic relationship will be related to lower ARA tendencies [1]. Based on the broader concept of Fraley’s “prototype model,” [40] which supports that earlier attachment experiences continue to exert influence over the years, a lengthy and relatively stable romantic involvement at the age of 16 may reduce ARA tendencies at the age of 18.

*H*_*3*_: It is expected that higher EIU behaviours will be related to higher ARA severity [48,55,57]. EIU may negatively influence ARA tendencies among adolescents, reinforcing fear of closeness and withdrawal from offline romantic interactions [48]. This is further supported by evidence that higher internet use is associated with the initiation of sexual activity prior to the involvement in a romantic relationship [42], which has been linked to ARA tendencies [14].

## Methods

### Participants

The present study received ethical approval from the ethics committee/ Institutional Review Board (IRB) of the National and Kapodistrian University of Athens, Greece. The implementation of the data collection required additional written permissions/approvals from: i) The Ministry of Education, ii) The Teachers’ Council, and iii) Parents’ consent. Specifically, parents/guardians had to sign a form that described the study aims, measures and processes and indicated their approval for their child to participate. The sample was selected from the Athens metropolitan area and a specific regional area using the method of randomized stratified selection based on the latest inventory card of the Ministry of Education (2010). The ratios of high schools and students were identified: 1) between the metropolitan area and the selected regional population and 2) between academic vs vocational track high schools. Based on these quotas participants were randomly (by lottery) selected at the classroom level.

The sample consisted of 515 Greek students embedded in 33 classrooms. Chi square analysis confirmed that the sample did not significantly differ from the original population regarding area of residence and the type of school of the participants χ^2^ = 1.58, DF 1,3, *p* = .66 (for exact quotas see Table 1). All participants in the study had internet access at time point 1 and 276 participants (53.6%) reported being involved in a romantic relationship at time point 1. With respect to parents’ and guardians’ socioeconomic profile, 80.2% were married, 6.2% of the mothers and 6.8% of the fathers were unemployed, and 77.9% of the mothers and 64.5% of the fathers had completed education equal to or above high school at time point 1. The estimated maximum sampling error with a size of 515 is 4.32% (z = 1.96. C.I = 95%).

**Table 1:** Original population and sample proportions.

				Area of Residence		
				Regional Area (Korinthia)	Athens Metro Area	Total
Population	Type of school	Vocational Track	N	744	13560	14304
			% of Total Population	0,83%	15,12%	15,95%
		Academic Track	N	2769	72614	75383
			% of Total Population	3,09%	80,96%	84,05%
		Total	N	3513	86174	89687
			% of Total Population	3,91%	96,08%	100%
Study Sample	Type of school	Vocational Track	N	7	49	56
			% of Total Sample	1,40%	9,50%	10,90%
		Academic Track	N	34	425	459
			% of Total Sample	6,60%	82,50%	89,10%
		Total	N	41	474	515
			% of Total Sample	8,00%	92,00%	100,00%

Note: Population refers to the relevant student population of the Athens Metro Area and the Regional Area (Korinthia).

Participants were assessed twice, two school years apart, and their responses were matched with a unique code (Wave 1: age *M* = 15.68 years, SD = 0.65, range 15.5–16.5, boys = 46.4%, girls = 53.6%; Wave 2: age *M* = 17.67 years, SD = .54, range 16.5–17.5, boys = 49.9%, girls = 50.1%). The retention rate was high (72%) with attrition due to changes of school, and school and research drop outs. To evaluate the attrition effects following Widaman [69], attrition was used as an independent variable (dummy coded 1 = Attrition, 0 = no attrition) at level 2 of the HLM analyses in order to assess whether it influenced ARA scores and its associations with the other independent variables. Results confirmed that attrition did not have any significant effects (Table 2).

**Table 2:** Assessment of the attrition effects in HLM analyses.

	Fixed Effects with Robust Standard Errors				
	* b *_*i*_	SE	T	*Df*	*P*_* i*_
Attrition	.07	.13	.51	32	.61
Gender * Attrition	.02	.17	.13	32	.90
Romantic Relationship * Attrition	-.31	.18	-1.76	32	.09
EIU * Attrition	.00	.01	.75	32	.46

Note: Attrition refers to participants who did not complete two measurements. To evaluate the attrition effects attrition was used as an independent variable (dummy coded 1 = Attrition, 0 = not attrition) at level 2 of the HLM analyses to assess whether it effects ARA behaviours and their associations with the other independent variables.

### Instruments

#### Experiences of Close Relationships Revised (ECR-R)

To assess ARA the relevant subscale of the ECR-R [70] was used. It consists of 36 self-report items that comprise two subscales, anxiety and avoidance, of 18 items each. Sample items include “I prefer not to show a partner how I feel deep down” (avoidance subscale). Participants answer each item on a 7-point Likert-type scale, where 1 = strongly disagree and 7 = strongly agree. The scores of all items are accumulated and the higher final score indicates stronger ARA tendencies. The ECR-R has been adapted in Greek and has been shown to have good psychometric properties [26,61]. In the present study, the instrument’s internal consistency was high with a Cronbach’s alpha of 0.85 for the avoidant attachment subscale.

#### Internet Addiction Test

The Internet Addiction Test (IAT) [71] was used to assess EIU. This scale has been validated in a sample of Greek adolescents [72]. The IAT includes 20 questions evaluating the importance of negative consequences because of EIU (e.g. “How often do you choose to spend more time on-line over going out with others?”). Questions 2, 3 and 8 were modified to reflect appropriate age/adolescence-related content (e.g., “How often does your job performance or productivity suffer because of the Internet?” was modified to be “How often does your school performance or productivity suffer because of the Internet?). There were six possible answers for each question on a Likert scale (0 = “it does not concern me” to 5 = “always”). The item scores are accumulated (0–100) and the higher final score indicates stronger EIU behaviours. The instrument’s internal consistency was high with a Cronbach alpha of 0.93.

#### Involvement in a romantic relationship

Participants were asked whether they were currently involved in a committed romantic relationship for more than three months. Following the definition of previous researchers [1], romantic relationships were described in this item as any kind of ongoing voluntary interaction that entailed a romantic commitment of particular intensity between two individuals of the same or the opposite sex (e.g., When you like a guy [girl] and he [she] likes you back). Such relationships were identified by expressions of affection and current or anticipated sexual behaviour (e.g. dating, holding hands, kissing, hugging). It is noted that the ethics approval received did not enable the use of a more specific romantic relationship scale.

### Procedures

The process of data collection was identical between the two time points. A specially trained research team of 13 undergraduate, postgraduate, and PhD students of the Department of Psychology of the University of Athens collected the data in the participants’ classrooms during the first two or the last two school hours (45 minutes each) of a school day.

### Analyses

Multilevel modelling was used to statistically analyse a data structure where measurements at two time points (level-1) were nested within individuals (level-2), who were nested within classrooms (level 3). This approach was chosen to enable us to disentangle and examine age related changes on ARA tendencies at level 1 and the effects of the two individual risks (i.e., being a male and presenting EIU behaviours) and the one resilience factor (i.e., involvement in a romantic relationship) on ARA tendencies at level 2, while controlling for possible random effects on ARA tendencies due to the nesting of the data (participants within classrooms) at level 3 (see S1 Table).

Conducting covariance based structural equation modelling (CBSEM) was not selected as: a) it requires at least three or four indicators (the current study includes two time points, which enable the examination of linear and not quadratic or cubic growth patterns [73]; for every latent variable (growth) [74] and; b) it assumes multi-normal distribution of the observed variables to ensure meaningful results-which is rarely the case in empirical research [75]. Similarly, latent growth modelling (LGM) was not chosen as it assumes that level-1 predictors with random effects have the same distribution across all participants in each subpopulation-while HLM allows different distributions [76]. Finally, HLM was preferred over partial least square analysis (PLS) and repeated measures ANOVA, as it estimates the effects of variables on the outcome variable at one level (i.e., individual), while at the same time considering the effect of variables on the outcome variable at another level (i.e., classroom) [76]. Subsequently, the HLM 6.0.8 software was used [77]. ARA symptoms (level-1 outcome variable) were predicted for each individual at Level 1 by wave in the study. Wave was centred at wave 1 such that the individual intercepts referred to the initial level of ARA (Wave 1 = 0, Wave 2 = 1). The individual initial level and the individual linear change over the two assessments (slope) were predicted at Level 2 by gender, involvement in a romantic relationship and EIU scores. It is noted that the last two variables varied across the period examined. However, given the emphasis of the present study on predictors of ARA tendencies, only time point one values were used as predictors on level 2. Level 1 was not an option given that wave was inserted as a level 1 variable and the HLM software can exclusively examine cross-level interactions [76]. This choice was based on recommendations that causal associations should be best addressed longitudinally [78]. Gender was centred on girls (0 = girls, 1 = boys) and involvement in a romantic relationship was centred on those who were not involved. To avoid co-linearity independent variables were inserted separately at level 2 (see S1 Table). Finally, the random effects due to the classrooms of the participants were controlled through random effects equations at level 3 in regard to both the main effects of time, as well as the cross-level interactions (slopes). The decision to control and not examine level 3 effects was based on two reasons: a) The theoretical emphasis of the current work on the “purer” effects of level 2 variables and; b) The insignificant contribution of level 3 on ARA tendencies’ variations (see results). To control for misspecification (i.e., lack of linearity) and the distributional assumptions at each level (lack of normality, heteroscedacity) HLM results accounting for robust standard errors (which are insensitive to possible violations of these assumptions) were calculated. At this point it should be noted that random effects calculated at the second and the third level may partially account for demographic individual and group characteristics not included as fixed effects in the present analyses (to the extent that the latter are reflected by the nesting of the data).

## Results

Prior to conducting the HLM analyses, the means and standard deviations of ARA and EIU at time point 1 and 2 were calculated. Further, ARA correlations with gender and engagement in a romantic relationship at the two time points were assessed (see Table 3).

**Table 3:** Means, standard deviations, correlations.

Wave	Mean	S D	1	2	3	4	5
1. ARA Wave 1	3.28	.91					
2. ARA Wave 2	3.15	.94	.49**				
3. EIU wave 1	29.53	17.55	.21**	.04			
4. EIU wave 2	24.31	17.99	.60**	.24**	.80**		
5. Gender	N/A	N/A	.07	-.07	.08	-.03	
6. Engagement in a romantic relationship	N/A	N/A	-.23**	.17**	.04	.13	.03

Note

* p≤ .05

** p≤ .01

***p≤ .001

Whereas missing values do not present a problem at level 1 (measurements within individuals) and did not occur at level 3 (classroom) which was controlled, they had to be addressed at level 2. Given that missing values were unsystematic and to avoid listwise deletion, multiple imputation was applied (five Maximum Likelihood imputations using SPSS) using all available Level 2 (individual) variables. This type of imputation was selected as it outperforms list-wise deletion for parameters involving many recouped cases and results to better standard error estimates [79,80]. Therefore, all equations were calculated five times, and the results were averaged in accordance with previous work [81]. Then models’ testing proceeded in successive phases [76]: unconstrained (null) model, random intercepts model (random ANCOVA model), means-as-outcome models separately for each level 2 predictor, and slopes as outcomes models (random regression model) separately for level 2 predictors, to avoid multicollinearity, and the full models. However, given that the results did not significantly differ, only the findings of the final models are reported here.

To assure that the multiple levels contributed to the overall variation, the variance components were calculated from the unconditional model (χ^2^ = 1034.9, df = 475, *p* < .001) and HLM equations were calculated (see S1 Table). The calculation of the intraclass correlation indicated that differences within individuals accounted for 39% of the variance of ARA, while differences between individuals accounted for 61% of the variance of ARA scores. Differences between classrooms of students accounted for less than 1% of the ARA variance and were controlled in the present analysis.

The Level 1 intercept for the cross-sectional findings was 3.29 representing the estimated average ARA score for adolescents at time 1. Regarding how ARA changes between 16 and 18 years (hypothesis 1), the time coefficient was *b* = -.15 (*p* = .007). This indicated that the average ARA score decreased to 3.14 (3.29-.15 = 3.14) at the age of 18. The addition of time in the model explained 3% of the ARA variance in level 1. The effect of gender on ARA (hypothesis 1) was insignificant both at the age of 16[*b* = .09, (*p* = .121)] and over time [*b* = .05, (*p* = .549)].

**Fig 1:**
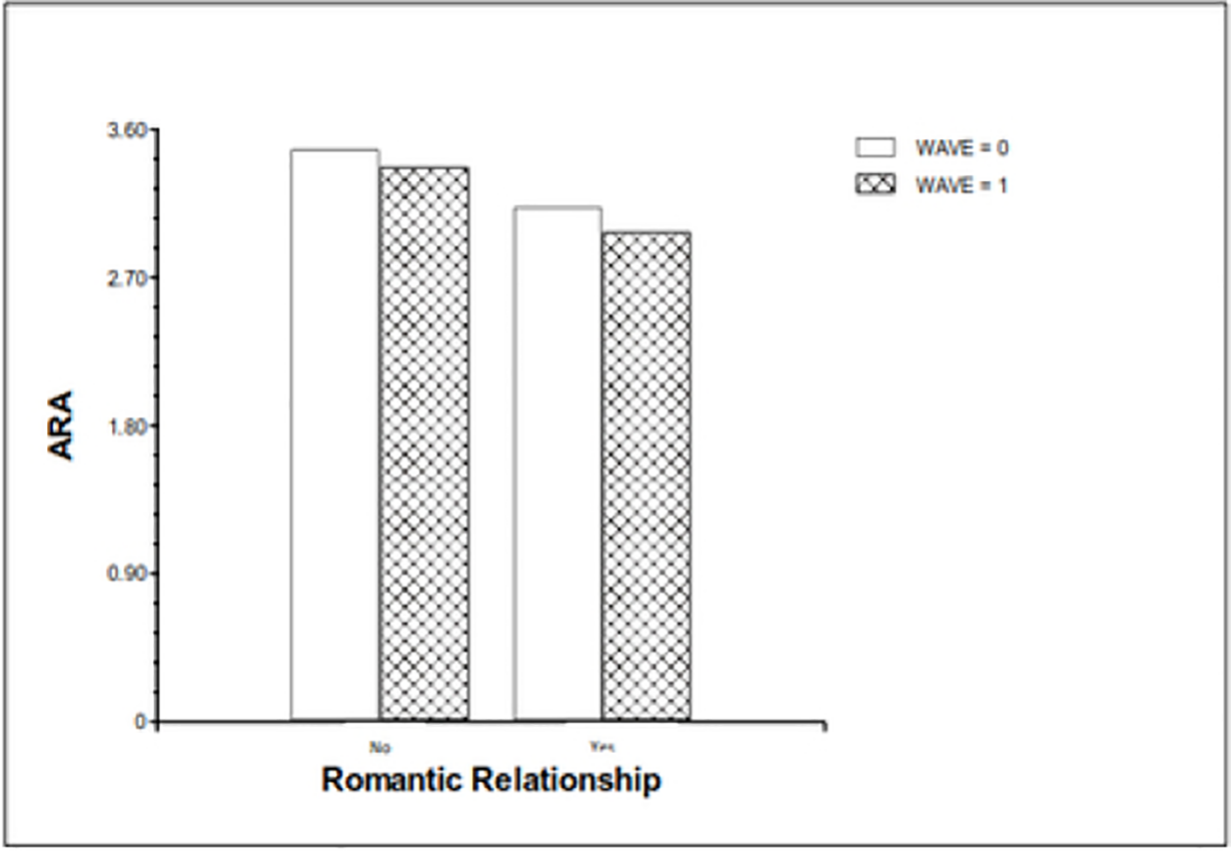
ARA and engagement in a romantic relationship at 16 and 18 years.

Engagement in a romantic relationship was negatively associated with ARA (hypothesis 2) [*b* = -.35, (*p* = .000)]. Consequently, the average ARA score of adolescents who were involved in a romantic relationship was lower at the age of 16. Engagement in a romantic relationship at the age of 16 did not significantly interact with time [*b* = -.04, (*p* = .588)]. It should be noted that the latter does not indicate that the association between engagement in a romantic relationship at the age of 16 and ARA tendencies at the age of 18 is insignificant. The finding indicates that the intensity of this association does not significantly differ from that of the association between engagement in a romantic relationship at the age of 16 and ARA tendencies at the same time (see Fig 1). The addition of engagement in a romantic relationship in the model explained 7% of the ARA variance in level 1 and 4% in level 2.

**Fig 2:**
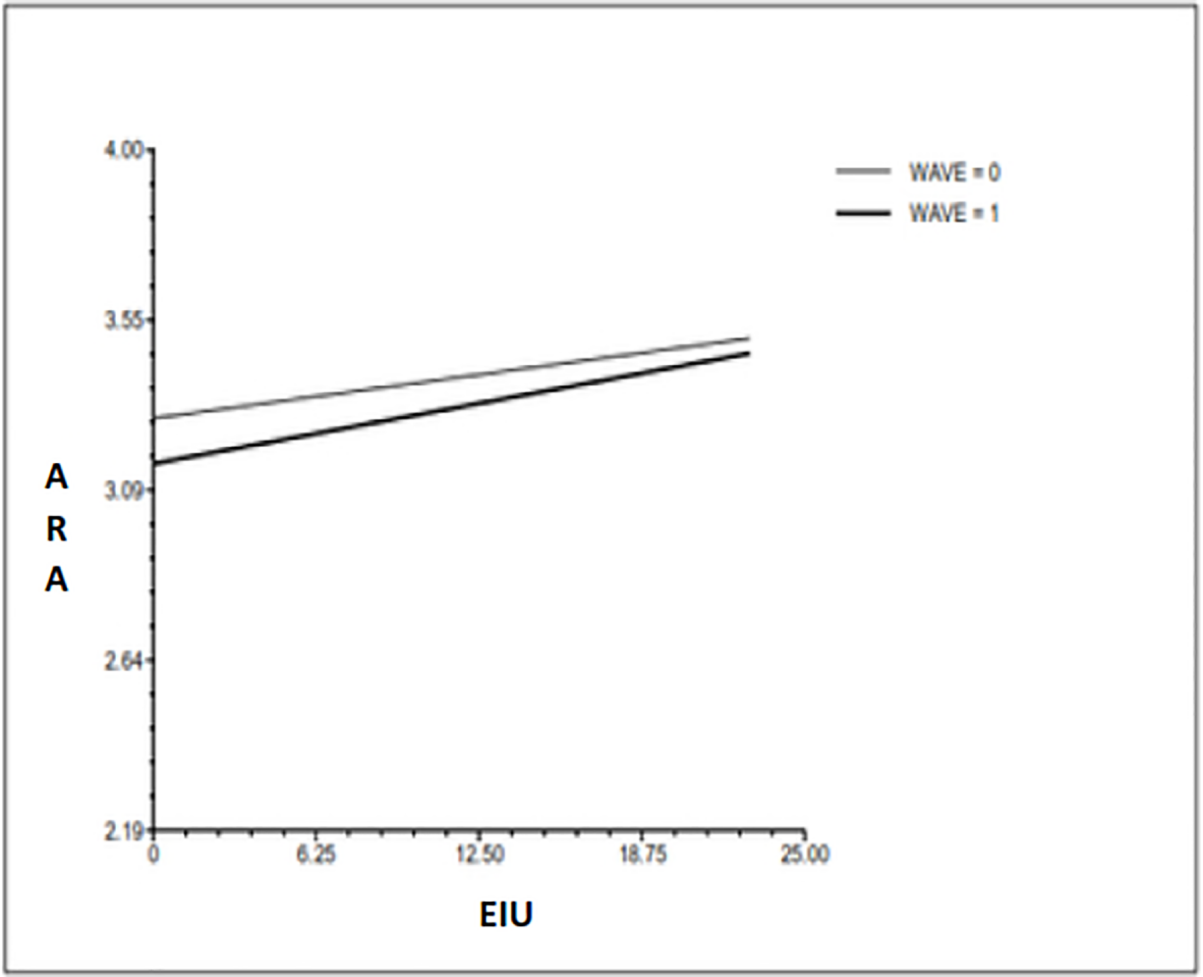
ARA and EIU at 16 and 18 years.

In terms of EIU levels in relation to ARA severity (hypothesis 3), the coefficient for EIU was *b* = .01 (*p* = .000) which indicates that an adolescent who scored one point higher on EIU than the average of adolescents at the age of 16 had an increased ARA score at the age of 16. However, the effect of the interaction of EIU scores with time was insignificant [*b* = .00, (*p* = .207)]. This reveals that the magnitude of the association between EIU at time point 1 and ARA tendencies at time point 2 does not significantly differ from that of the association between EIU at time point 1 and ARA tendencies at time point 1. The addition of EIU scores in the model explained 5% of the ARA variance in level 1 and 6% of the variance in level 2 (see Fig 2). All analyses controlled for random effects at levels 1, 2 & 3 (see Table 4).

**Table 4:** HLM analysis predicting adolescents’ ARA scores.

* *	*Cross-sectional Results*				
* *	*Fixed Effects with Robust Standard Errors*				
* *	*b*_*i*_	*SE*	*T*	DF	*p*_*1*_
Intercept	3.29	.04	86.24	32	.000
Gender	.09	.06	1.59	32	.121
Romantic Relationship	-.35	.08	-4.56	32	.000
EIU	.01	.00	4.27	32	.000
* *	*Over time results*				
* *	*Fixed Effects with Robust Standard Errors*				
* *	*b*_*i*_	*SE*	*T*	DF	*p*_*1*_
Intercept (Time)	-.15	.05	-2.91	32	.007
Gender	.05	.09	.61	32	.549
Romantic Relationship	-.04	.08	-.55	32	.588
EIU	.00	.00	1.29	32	.207

Note: The table summarizes the main results regarding the individual factors examined and is divided into two parts. The upper part presents the cross-sectional findings after controlling for random effects at Levels 2 (individual) and 3 (classroom). The lower part presents the over-time change results after controlling for random effects at Levels 2 (individual) and 3 (classroom).

## Discussion

In the present study an integrative, multilevel approach that combined the attachment literature and the risk and resilience framework was adopted to examine the longitudinal variations in ARA severity in a normative sample of Greek adolescents assessed longitudinally at 16 and 18 years of age. Specifically, the aim was to examine age-related change in ARA behaviours between 16 and 18 years taking into consideration the effects of individual level risks and resources, while controlling for other clustering (individual and classroom) effects. This integrative framework was operationalized via a multi-level hierarchical linear model. The model was composed of three levels: the temporal factors (i.e., ARA over time), being male and presenting with EIU behaviours as individual level risks and the engagement in a romantic relationship as an individual level resource. Random effects due to the clustering of the participants were controlled at levels 2 and 3. ARA decreased between 16 and 18 years. Furthermore, adolescents reporting involvement in a committed romantic relationship were significantly lower on ARA at the age of 16 years and this association did not significantly vary over time. Students at age 16 reporting higher EIU behaviours presented with higher ARA tendencies and this effect was not significantly moderated by age. Finally, gender was not found to differentiate ARA scores neither at the age of 16 nor over time.

### Age related changes in understanding, prevention and treatment of ARA tendencies

To the best of the authors’ knowledge, there is a dearth of longitudinal studies investigating the effect of age related changes on ARA severity especially. Previous cross-sectional findings based on comparisons of different age groups have contended that ARA severity varies over the life course, with a tendency to increase after the age of 20, in particular in males, and to stabilize sometime after the age of 40 [14]. Our finding appears to be in consensus with previous studies that have found fluctuations of the severity of ARA tendencies over time, as well as a tendency of secure attachment to increase for individuals, not exposed to risk factors [14,20,21], during late adolescence. Furthermore, our findings extend previous research by providing knowledge in relation to a culturally under-researched sample of Greek adolescents.

The present finding supports that ARA tendencies decrease between 16 and 18 years in a representative sample of adolescents in Greece. Therefore, one could assume that ARA tendencies may not steadily increase over time, similar to the period between 20 and 40 years of age [14], but they may rather fluctuate depending on the developmental stage of the individual. This interpretation is consistent with the risk and resilience framework, which assumes that behaviours presenting as problematic, may often have developmental characteristics related to normative changes over the lifespan [62]. This may reinforce the possibility of a developmental change to lower ARA tendencies during the period of late adolescence [3]. This potential explanation appears consistent with the effects of cognitive, emotional and identity maturation during late adolescents, that may provide adolescents with the skills needed to feel more comfortable with romantic intimacy [1,15,16]. Furthermore, ARA tendencies may tend to fade due to the effects of nervous system maturation, for example, higher sympathetic nervous system reactivity and higher parasympathetic nervous system withdrawal that enrich adolescents with more effective stress coping strategies [17–19]. Nevertheless, the present findings contradict past studies which have revealed a general stability of attachment styles during adolescence in other national samples. This could indicate a (culturally) specific tendency of ARA behaviours to decrease among adolescents in Greece between 16 and 18. This decrease could be attributed to Greek traditional values favouring stability, as well as the institutions of marriage and family [1,27]. Given that: a) this is the first prospective study of ARA severity in adolescents in Greece during the period between 16 and 18 years and; b) the sample in the present study included exclusively high school students and not late adolescents who were not attending lyceum/secondary high school and therefore, may experience different patterns of ARA development, this conclusion needs to be interpreted with caution. Despite the need for more longitudinal and cross-cultural studies of typical adolescent development between 16 and 18 years, results have direct implications for the planning of prevention and treatment initiatives. Specifically, they highlight: (i) the need for more prevention resources and programs to be focused on adolescents after the age of 18 years in order to prevent age-related ARA behaviours from escalating into clinical problems later in life—especially for more vulnerable individuals; and (ii) the need for intervention and treatment programs to target individuals who present with more severe ARA tendencies after 18 years, posing a potential clinical risk.

### Gender in understanding, prevention and treatment of ARA tendencies

The findings of this study did not reveal any gender associated variations in ARA severity between 16 and 18 years in the sample of Greek adolescents examined. This finding appears to contradict previous literature that has found males to report higher ARA behaviours than females [14,28]. However, our results are in line with findings that propose that gender related variations in ARA behaviours appear to be associated with age related changes, as well as cultural dimensions [14,28]. Specifically, past studies have supported that gender differences in romantic attachment attenuate in student samples due to homogeneity reasons such as educational and social compliance effects [14]. Furthermore, gender-related differences in facets of ARA including “self-reliance” and “discomfort with closeness” have been reported to fluctuate across different cultural samples (US, Italian) [28]. Based on this, there are two explanations that may account for the present finding. First, it may be that gender related differences in ARA emerge later than the age of 18 or that there are cultural effects that minimise such differences in Greek samples in particular. It is likely that the strong collectivistic and family orientation of the Greek culture weakens gender differences in ARA severity, which is associated with more transient and less committed romantic involvement [27–29]. The latter is reinforced by previous cross-sectional findings from cross-cultural studies, indicating that: a) Greek samples did not present significant gender differences in ARA [25] and; b) that gender specific ARA differences minimize in contexts where the reproductive interests of males and females converge [14].

Given the nature of this finding, further research is required to assess whether it is indicative of age related/ developmental effects, cultural dimensions or a combination of both that results in no significant gender difference in ARA tendencies during late adolescence. Despite these limitations, directions for ARA prevention and treatment are implied. Prevention initiatives may not need to be gender-specific for Greek adolescents during the period between 16 and 18 years. Furthermore, treatment of ARA tendencies among Greek adolescents should embrace cultural elements that emphasize orientation to more committed and stable relationships.

### Involvement in a romantic relationship in understanding, prevention and treatment of ARA tendencies

The findings suggested that a concurrent involvement in a committed romantic relationship for more than three months functioned as an ARA resource. This is in line with previous literature supporting the significance of engagement in romantic relationships for adolescents [1,30,32]. Furthermore, it is reinforced by research indicating the positive behavioural impact on general adaptation that results from the engagement of adolescents in romantic relationships [31]. The current findings further compliment recent studies examining involvement in romantic relationships as a factor predicting ARA [36,37]. The experience of a functional romantic relationship may reduce concerns and fears of rejection that cause discomfort and avoidance, contributing to the reduction of ARA tendencies in adolescence. On the contrary, the involvement in a relatively lengthy and stable/committed romantic relationship at the age of 16 could precipitate and perpetuate positive expectations that may decrease ARA behaviours overtime. This interpretation is in accordance with studies suggesting that past experiences of romantic relationships define internal representations that could precipitate future romantic behaviour and impact ARA severity [36,37]. Furthermore, this explanation aligns with the broader concept of Fraley’s “prototype model,” [70] which supports that earlier attachment experiences continue to exert influence over the years. Such positive experiences of relationships could be more beneficial between 15 and 18 years of age, when important shifts occur with respect to intimacy and interdependence [1,3].

Nevertheless, the present finding contradicts research supporting that the engagement in romantic relationships in adolescence is associated with rather negative behavioural outcomes which may include higher delinquent behaviours, signs of depression and suicidality [33,34]. This contradiction could be explained by the type of questions asked of the participants. Specifically, in the present study participants were asked about whether they participated in a romantic relationship with specific features/characteristics. These involved a relationship duration that exceeded three months and a sense of commitment. Interestingly, past studies have concluded that romantic relationships in adolescence could operate both as risks and as resources based on qualities such as time length and commitment of the romantic interaction that the adolescents engage in [35]. Therefore, it is likely that the nature of the questions posed here may have targeted more functional and adaptive romantic relationships that tend to have positive effects on adolescents’ romantic development and not romantic relationships in general [1]. This finding expands the existing knowledge in the field by highlighting the positive effect of involvement in romantic relationships at the age of 16 on ARA tendencies. Further study is required in relation to the specific qualities of romantic relationships and the way these could impact adolescents’ ARA behaviours later in development.

Despite these limitations, our results emphasize the need for accounting for involvement in current romantic relationships’ when planning ARA prevention and intervention initiatives. In particular, in line with existing practices, ARA prevention programs in adolescence could benefit from embracing attachment oriented tasks that emphasize on the significance of romantic relationships and the development of romantic relational strategies [82]. These could be achieved through the implementation of behavioural observation, reflection, role playing and role modelling techniques [82]. In that context, the introduction of alternative romantic expectations, along with the exposure to material that may confront established romantic beliefs may generate the opportunity for personal re-evaluation and change [82].

### EIU in understanding, prevention and treatment of ARA tendencies

EIU behaviour was found to be an ARA risk at 16 years and the strength of this association did not change over time. This finding is in accordance with previous studies highlighting the significant impact of internet use on several developmental milestones in adolescence, including romantic development [43]. Accordingly, it adds to literature highlighting how the use of social networking sites might be compensating for social skills difficulties related to face to face communication [83–85] by paradoxically enabling individuals to experience closeness whilst being distant [86]. In that context, the present study further highlights the links between avoidant tendencies and EIU in particular [49–51]. Finally, it potentially aligns with available knowledge considering how internet use could compromise sexual and romantic development in adolescence [45,46].

A potential interpretation of this finding could be that adolescents with EIU behaviours progressively become more dependent on the flexibility and convenience of cyber-interactions factors such as anonymity, accessibility, affordability, and without cultivating the face to face social skills that may enable them to feel more comfortable with romantic intimacy in real life and therefore, present higher ARA tendencies [48,55]. This explanation is reinforced by studies contending that behaviours are calibrated by functionality requirements according to the contextual conditions which tend to be more convenient in the internet context [14,57]. Accordingly, EIU may negatively influence ARA tendencies among adolescents, fostering fear of closeness and withdrawal from offline romantic interactions as ways of addressing the challenges of real-life romantic relationships [48,54]. This interpretation is in line with the *“social compensation hypothesis”* proposed by Desjarlais and Willoughby [47].

Furthermore, it needs to be noted that the present finding refers to the effect of EIU behaviours on ARA severity and not the effect of internet use in general. The effect of internet use on romantic behaviour can be both *“debilitating and/or deliberating”* depending on its specific qualities for example, the time spent online, type of motivation, individual differences regarding the users’ profiles and the applications used [48]. In that context, research has suggested potential favourable effects of internet use [43,44]. Considering the present finding in particular, it could be assumed that the *“rich-get-richer”* hypothesis does not apply to individuals reporting high EIU behaviours [45]. EIU behaviours are intertwined with psychopathological symptoms and disadvantages or delays in regard to the achievement of developmental tasks [50]. Overall, the present finding contributes to the extant knowledge on the topic by revealing the risk effect of EIU on ARA severity during the period of late adolescence.

Although further longitudinal and cross-cultural studies are required to support these explanations, directions for ARA prevention and treatment are implied. Prevention initiatives should consider Internet use behaviours and emphasize the higher risk of adolescents presenting with EIU behaviours. For individuals presenting with EIU in particular, treatment of ARA tendencies should target internet overuse, decreasing FtF relationship avoidance, especially during the transitional time between adolescence and young adulthood.

### Limitations, significance & further research

Our measurements were based on self-report questionnaires. Furthermore, beside its factual quality, its compliance with the ethical approval provided by the Greek ministry of education and its consensus with previous theoretical definitions [1], romantic relationship involvement was assessed with a binary question. This may not have provided the chance for a wider range of answers and variations to be recorded. Similarly, considering the measurement section, it is likely that partner identification may have impacted the validity of ECR-R scores for adolescents without previous experience in romantic partnerships. Moreover, significant changes to the frequency, the accessibility, and the patterns of Internet use during adolescence may have occurred since 2010 and 2012 that the data was collected. Specifically, it should be highlighted that smart phones have become more common place enabling easier adolescent Internet access, and potentially without parental permission. In that context, the IAT items (created in 1998) may not accurately represent current Internet use. The above sampling and measuring restrictions introduce caution to the usability of the findings and illustrate the need for future investigation. In addition, our sample comes from the Greek cultural context and was measured only twice within a very specific (although critical) developmental period. Therefore, findings cannot be generalized across the lifespan or across different cultures. In that line, the present study emphasized exclusively the causative effect of EIU on ARA behaviours, while this association presents to have been bi-directional [54–56]. Subsequently, relevant considerations need to be taken into account when interpreting the relationship between the two behaviours. Finally, effects of demographic characteristics were not directly controlled in the analyses conducted.

Despite these limitations, our study has several significant strengths. These include: (i) the longitudinal design; (ii) the target population’s representativeness; (iii) the three-level hierarchical model applied that enabled us to disentangle individual level effects on ARA patterns, controlling for other individual and classroom level random effects; (iv) the use of a non-US sample, thus expanding the corpus of nations in which attachment is studied; (v) the emphasis on an under-researched cultural population; and (vi) the innovative empirical findings considering the effects of age related changes, gender, involvement in romantic relationships and EIU on ARA development.

The present study has implications for prevention and intervention initiatives in relation to the romantic development of adolescents and further research. Interestingly, and in contrast with past literature [14,28], no gender associated variations in ARA severity were revealed. Given the specific age and cultural aspects of the present sample, the need for further research considering the potential effects of age related changes and cultural dimensions [14,28] in ARA behaviours is illustrated. Similarly, the need of cross-lagged analyses studies to clearer describe the bi-directional association between ARA and EIU behaviours is underpinned by the present findings. In terms of treating ARA risk in adolescence, it appears that initiatives should focus on individuals who are not involved in romantic relationships and present EIU behaviours, especially if ARA severity persists after the age of 18 years.

Interestingly, interventions should include equipping adolescents with FtF communication skills that would reduce the need for online avoidant tendencies and amplify romantic involvement. Such interventions could be combined with advancements in applied Attachment Based Therapies (ABT) [80,87]. For example, ABT interventions that target the Internal Working Models (IWMs-representations) of relationships of adolescents by providing them restorative experiences, such as the “Circle of Security” (COS) program [88] could be appropriately modified. Specifically, to help adolescents revise their potentially ARA related dysfunctional IWMs of romantic relationships, therapeutic conversations at the individual or group level could be utilized. These could embrace already applied ABT techniques by targeting on (1) ARA narratives; (2) identifying, naming, and validating feelings that are interwoven with ARA narratives; and (3) restructuring ARA expectations into more secure IWMs [89]. These initiatives are particularly important in the light of the well-established negative associations between ARA tendencies and general satisfaction, connectedness, and general support in relationships [4].

Finally, the present study suggests that ARA tendencies in adolescence could constitute a transient developmental behaviour and should not be viewed as pathological. In that context, the results address and simultaneously reinforce previous research recommendations that support the need of adopting a longitudinal and contextualized approach when studying romantic development in adolescence, and aim to treat less desirable romantic attachment behaviours [1,3,14,28].

## Compliance with ethical standards

### Ethical standards–animal rights

All procedures performed in the study involving human participants were in accordance with the ethical standards of the institutional and/or national research committee and with the 1964 Helsinki declaration and its later amendments or comparable ethical standards. This article does not contain any studies with animals performed by any of the authors.

### Informed consent

Informed consent was obtained from all the parents and the guardians of the adolescents participating in the study, as well as the individual participants themselves.

## Supporting information

S1 Table Specification of the multilevel analyses for the three research hypotheses. (DOCX)
