# Supplementary material for: Avoidant romantic attachment in adolescence: Gender, excessive internet use and romantic relationship engagement effects
Source: PLoS One. 2018 Jul 27;13(7):e0201176. doi: 10.1371/journal.pone.0201176 (PMC6063419; doi:10.1371/journal.pone.0201176)
Supplement: S1 Table — (DOCX) [file pone.0201176.s001.docx]

**Supporting Information**

|  |  |  | *Hypotheses* |  |
| --- | --- | --- | --- | --- |
|  | *H_1a_* | *H_1b_* | *H_2_* | H_3_ |
| Level 1 | *ECR-R Avoidance = π_0_+ π_1_*(WAVE) + ε* | *ECR-R Avoidance = π_0_+ π_1_*(WAVE) + ε* | *ECR-R*  *Avoidance*  *= π_0_+*  *π_1_*(Wave) + ε* | *ECR-R*  *Avoidance*  *= π_0_+*  *π_1_* (Wave) + ε* |
| Level 2 | *π_0_ = β_00_ + ρ_0_* | *π_0_ = β_00_ + β_01_*(sex) + ρ_0_* | *π_0_ = β_00_ + β_01_*(Romantic Relationship) + ρ_0_* | *π_0_ = β_00_ + β_01_*(IA) + ρ_0_* |
|  | *π_1_ = β_10_ + ρ_1_* | *π_1_ = β_10_ + β_11_*(sex) + ρ_1_* | *π_1_ = β_10_ + β_11_*(Romantic Relationship) + ρ_1_* | *π_1_ = β_10_ + β_11_*(IA) + ρ_1_* |
| Level 3 | *β_00_ = γ_000_ + u_00_* | *β_00_ = γ_000_ + u_00_* | *β_00_ = γ_000_+ u_00_* | *β_00_ = γ_000_ + u_00_* |
|  | *β_01_ = γ_010_ + u_01_* | *β_01_ = γ_010_ + u_01_* | *β_01_ = γ_010_ + u_01_* | *β_01_ = γ_010_ + u_01_* |
|  | *β_10_ = γ_100_ + u_10_* | *β_10_ = γ_100_ + u_10_* | *β_10_ = γ_100_ + u_10_* | *β_10_ = γ_100_ + u_10_* |
|  | *β_11_ = γ_110_ + u_11_* | *β_11_ = γ_110_ + u_11_* | *β_11_ = γ_110_ + u_11_* | *β_11_ = γ_110_ + u_11_* |

**S.1 Table. Specification of the Multilevel Analyses for the Three Research Hypotheses.**
